# Cats learn the names of their friend cats in their daily lives

**DOI:** 10.1038/s41598-022-10261-5

**Published:** 2022-04-13

**Authors:** Saho Takagi, Atsuko Saito, Minori Arahori, Hitomi Chijiiwa, Hikari Koyasu, Miho Nagasawa, Takefumi Kikusui, Kazuo Fujita, Hika Kuroshima

**Affiliations:** 1 Department of Psychology, Graduate School of Letters, Kyoto University, Yoshida-honmachi, Sakyo, Kyoto, 606-8501 Japan; 2 Department of Animal Science and Biotechnology, Azabu University, 1-17-71, Fuchinobe, Chuo-ku, Sagamihara, Kanagawa 252-5201 Japan; 3 Japan Society for the Promotion of Science, 5-3-1, Chiyoda-ku, Tokyo, 102-0083 Japan; 4 Department of Psychology, Faculty of Human Sciences, Sophia University, 7-1, Kioicho, Chiyoda-ku, Tokyo, 102-8554 Japan; 5 Research and Development Section, Anicom Speciality Medical Institute Inc., 2-6-3 Chojamachi 5F, Yokohamashi-Nakaku, Kanagawaken, 231-0033 Japan; 6 Wildlife Research Center, Kyoto University, 2-24 Tanaka-Sekiden-cho, Sakyo, Kyoto, 606-8203 Japan

**Keywords:** Developmental biology, Psychology, Zoology

## Abstract

Humans communicate with each other through language, which enables us talk about things beyond time and space. Do non-human animals learn to associate human speech with specific objects in everyday life? We examined whether cats matched familiar cats’ names and faces (Exp.1) and human family members’ names and faces (Exp.2). Cats were presented with a photo of the familiar cat’s face on a laptop monitor after hearing the same cat’s name or another cat’s name called by the subject cat’s owner (Exp.1) or an experimenter (Exp.2). Half of the trials were in a congruent condition where the name and face matched, and half were in an incongruent (mismatch) condition. Results of Exp.1 showed that household cats paid attention to the monitor for longer in the incongruent condition, suggesting an expectancy violation effect; however, café cats did not. In Exp.2, cats living in larger human families were found to look at the monitor for increasingly longer durations in the incongruent condition. Furthermore, this tendency was stronger among cats that had lived with their human family for a longer time, although we could not rule out an effect of age. This study provides evidence that cats link a companion's name and corresponding face without explicit training.

## Introduction

Many human words have referential meanings: they evoke a visual mental image when heard or read^1^. For example, the word “apple” causes us to imagine a red or green fruit even if no such fruit is present. This language property, which expands the plasticity of communication, is also seen to some extent in non-human animals, mainly in the context of intraspecific vocal communication. Seyfarth, Cheney and Marler reported that vervet monkeys (now called *Chlorocebus pygerythrus*) responded differently to different types of alarm calls^2^ (although some of the calls overlap acoustically^3^ and this view is currently debated^4^). More recently, west African green monkeys (*Chlorocebus sabaeus*) rapidly learned the novel referent of an alarm call that was given in response to a drone^5^. Referential signaling is not limited to primates. Suzuki showed that tits (*Parus minor*) detected snake-like motion more rapidly when a snake-specific alarm call rather than a general alarm call was played back, suggesting that tits recall things to which at least one specific call refers^6^. Such studies show that animals have specific calls with a referential meaning, increasing the likelihood of responses appropriate for survival.

In contrast to studies dealing with life-or-death-related issues and ecology, some studies have reported that companion animals understand human utterances in more neutral situations and use them in communication with us [e.g., dogs (*Canis lupus familiaris*)^7–12^]. Dogs in particular have been studied in this context; for example, a few “expert” dogs trained in object-name fetching over several months remembered hundreds of object names and fetched the correct object upon verbal command^7,8,12^. According to a recent report, “gifted” dogs learned object names after few exposures during social interactions, whereas the majority of dogs did not show such object-name association learning despite intensive training^12^.

Similar to dogs, cats (*Felis catus*) are one of the most widespread companion animals in the world^13^ . Although the ancestral Libyan wildcat (*Felis lybica*) is a solitary species^14^, many domestic cats live with humans and show evidence of social cognitive operations concerning humans. They can use human pointing cues^15^ and gaze cues^16^ to find food. They also discriminate between human facial expressions^17–19^ and attentional states^20–22^, and identify their owner’s voice^23^. Furthermore, they cross-modally match their owner's voice and face^24^ when tested with their owner’s photo presented on a screen, and human emotional sounds and expressions^19^.

Cats have been shown to distinguish their own from another familiar cat’s name in a habituation–dishabituation procedure^25^, and they also distinguished those names from general nouns. Interestingly, cats living in multi-cat households habituated less to their companion cats’ names than to other nouns. Conceivably, therefore, cats might also recognize the name of another cat living in the same household.

Here we examined whether cats linked a human utterance and the corresponding object, using a relatively simple task that is applicable to many species: a visual-auditory expectancy violation task previously used to test cats’ ability to predict an object when hearing that objects’ name^24^. As stimuli we used the names of other cats (“models”) cohabiting with the subjects in Exp.1, and human family members’ names in Exp.2. Cats were presented with the face of the other cat (Exp.1) or human (Exp.2) following presentation of the model’s name, called by the owner (Exp.1) or an experimenter (Exp.2). Half of the trials were “congruent,” i.e., the model’s face and name matched, whereas the other half were “incongruent” (the stimuli mismatched). Previous research showed that cats matched human photos and voices^24^, which established the validity of presenting photos as stimuli. Our hypothesis was that cats learned face–name relationships by observing interactions involving their owner, and that more such observations would lead to stronger learning. We tested two groups of cats, differing in the number of other cats they lived with: cats belonging to cat cafés where many cats live together, and household cats. The latter probably have more opportunities to observe interactions between the owner and each of the other cohabitating cats, which might facilitate learning of the face–name relationship. Therefore, we analyzed data from household cats and cat café cats separately in Exp.1. In Exp.2, analysis concerned the number of cohabiting family members because more members would have more opportunities to hear other members’ names (e.g., people living as a couple probably say each other’s name less often than people living in a larger family). In Exp.2 we considered length of time living with the family as well as the number of family members.

We made two predictions. First, attention toward the stimulus face displayed on the monitor should be longer in incongruent trials due to expectancy violation. Second, the amount of violation is related to the amount of exposure to relevant interactions; specifically, household cats should show stronger violation effects than café cats in Exp.1, and cats living in households with more people should show more evidence of expectancy violation in Exp.2.

## Experiment 1

### Materials and methods

#### Subjects

We tested 48 cats (28 males and 19 females). Twenty-nine (17 males and 12 females, mean age 3.59 years, *SD* 2.71 years) lived in five “cat cafés” (mean number living together: 14.2, *SD* 10.01), where visitors can freely interact with the cats. The other 19 (11 males and 8 females, mean age 8.16 years, *SD* 5.16 years) were household cats (mean number living together: 6.37, *SD* 4.27). We tested household cats living with at least two other cats because the experiment required two cats as models. The model cats were quasi-randomly chosen from the cats living with the subject, on condition of a minimum period of 6 months cohabiting, and having different coat colors so that their faces might be more easily identified. We did not ask the owner to make any changes to water or feeding schedules.

#### Stimuli

For each subject, visual stimuli consisted of two photos of two cats other than the subject who lived together, and auditory stimuli consisting of the voice of the owner calling the cats’ names. We asked the owner to call each cat’s name as s/he would usually do, and recorded the call using a handheld digital audio recorder (SONY ICD-UX560F, Japan) in WAV format. The sampling rate was 44,100 Hz and the sampling resolution was 16-bit. The call lasted about 1 s, depending on the length of cat’s name (mean duration 1.04 s, *SD* 0.02). All sound files were adjusted to the same volume with the help of version 2.3.0 of Audacity(R) recording and editing software^26^. We took a digital, frontal face, neutral expression, color photo of each cat against a plain background (resolution range: x = 185 to 1039, y = 195 to 871) which was expanded or shrunk to fit the monitor size (12.3″ PixelSense™ built-in display).

#### Procedure

We tested cats individually in a familiar room. The cat was softly restrained by Experimenter 1, 30 cm in front of the laptop computer (SurfacePro6, Microsoft) which controlled the auditory and visual stimuli. Each cat was tested in one session consisting of two phases. First, in the name phase the model cat’s name was played back from the laptop’s built-in speaker four times, each separated by a 2.5-s inter-stimulus interval. During this phase, the monitor remained black. Immediately after the name phase, the face phase began, in which a cat's face appeared on the monitor for 7 s. The face photos were ca. 16.5 × 16 cm on the monitor. Experimenter 1 gently restrained the cat, looking down at its head; she never looked at the monitor, and so was unaware of the test condition. When the cat was calm and oriented toward the monitor, Experimenter 1 started the name phase by pressing a key on the computer. She restrained the cat until the end of the name phase, and then released it. Some cats remained stationary, whereas others moved around and explored the photograph presented on the monitor. The trial ended after the 7-s face phase.

**Figure 1:**
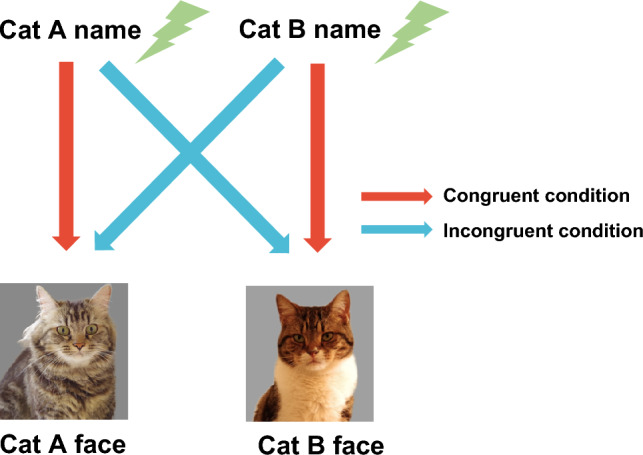
Diagram illustrating each condition in Exp.1. Two model cats were chosen from cats living with subject. The model cat’s name called by owner was played through the speaker built into the laptop computer (Name phase). Immediately after playback, a cat’s face appeared on the monitor (Face phase). On half of the trials the name and face matched (congruent condition), on the other half they mismatched (incongruent condition).

We conducted two congruent and two incongruent trials for each subject (Fig. 1), in pseudo-random order, with the restriction that the same vocalization was not repeated on consecutive trials. The inter-trial interval was at least 3 min. The subject’s behaviors were recorded on three cameras (two Gopros (HERO black 7) and SONY FDR-X3000): one beside the monitor for a lateral view, one in front of the cat to measure time looking at the monitor, and one recording the entire trial from behind.

#### Analysis

One cat completed only the first trial before escaping from the room and climbing out of reach. For the face phase we measured time attending to the monitor, defined as visual orientation toward or sniffing the monitor. Trials in which the subject paid no attention to the monitor in the face phase were excluded from the analyses. In total, 34 congruent trials and 33 incongruent trials for café cats, and 26 congruent trials and 27 incongruent trials for house cats were analyzed (69 trials excluded overall). A coder who was blind to the conditions counted the number of frames (30 frames/sec.) in which the cat attended to the monitor. To check inter-observer reliability, an assistant who was blind to the conditions coded a randomly chosen 20% of the videos. The correlation between the two coders was high and positive (Pearson’s *r* $=$ 0.88, *n* $=$ 24, *p* < 0.001).

We used R version 3.5.1 for all statistical analyses^27^. Time attending to the monitor was analyzed by a linear mixed model (LMM) using a lmer function in a lme4 package version 1.1.10^28^. We log-transformed attention time to get close to normal distribution. Congruency (congruent/ incongruent), environment (cat café/house), and the interaction were entered as fixed factors, and subject identity was a random factor. We ran F tests using an Anova function in a car package^29^ to test whether effects of each factor were significant. To test for differences between conditions, an emmeans function in an emmeans package^30^ was used, testing differences of least squares means. Degrees of freedom were adjusted by the Kenward–Roger procedure.

In addition to attention to the monitor, we calculated the Violation Index (VI), which indicates how much longer cats attended in the incongruent condition than the congruent condition. VI was calculated by subtracting the mean congruent value from the mean incongruent value for each subject. Greater VI values indicate longer looking in incongruent conditions. Note that we used data only from subjects with at least one congruent—incongruent pair. Thus, if a subject had one congruent/incongruent data point, we used that value for analysis instead of calculating the mean. Data from 14 household cats and 16 café cats were analyzed. We ran a linear model (LM) using a lmer function in a lme4 package version 1.1.10^28^. Living environment (café/house) was entered as a fixed factor. To examine whether VI was greater than 0, we also conduct a one-sample t-test for each group.

### Results and discussion

**Figure 2:**
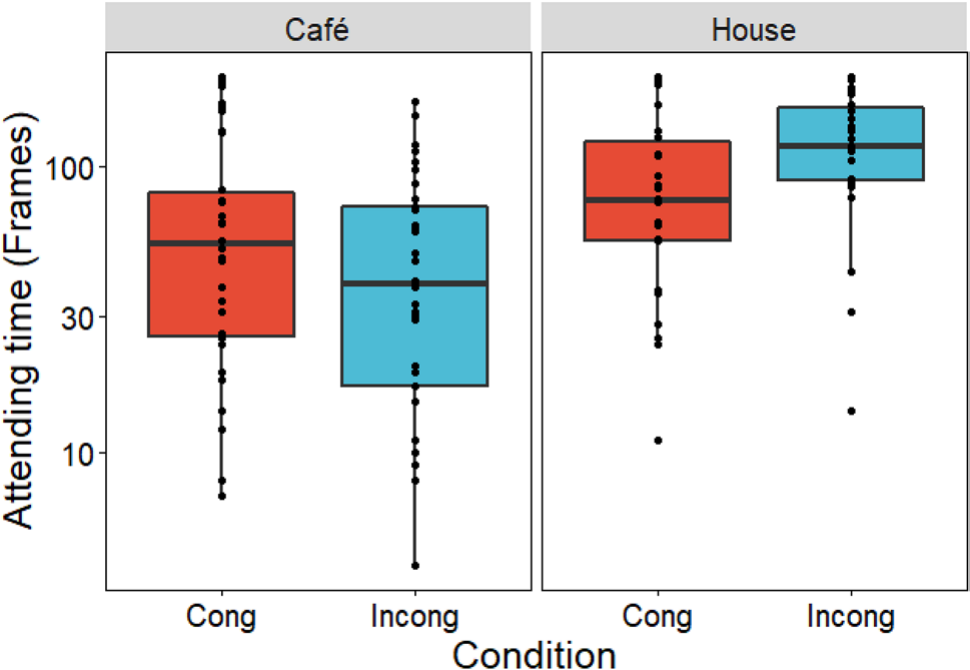
Time attending to the monitor during the face phase for each group in Exp.1. Red bar represents congruent condition; Blue bar represents incongruent condition. Left panel shows café cat data, right panel shows house cat data. The y-axis is log-transformed.

Figure 2 shows time attending to the monitor for each group. House cats attended for longer in the incongruent than the congruent condition, as predicted; however, café cats did not show this difference.

LMM revealed a significant main effect of living environment (${\rm X}$^2^ (1) = 16.544, *p* < 0.001), and a congruency x living environment interaction (${\rm X}$^2^ (1) = 6.743, *p* = 0.009). The differences of least squares means test confirmed a significant difference between congruent and incongruent conditions in house cat (*t* (86) = 2.027, *p* = 0.045), but not café cats (*t* (97.4) = 1.604, *p* = 0.110).

**Figure 3:**
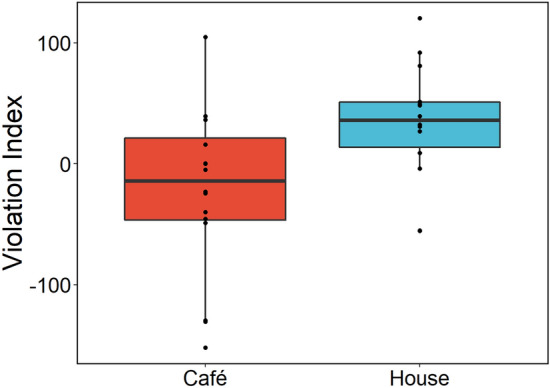
Violation Index for each group in Exp.1. Red boxplot (left) shows café cat data; blue boxplot (right) shows house cat data.

Figure 3 shows the difference in VI between groups. House cats had a significantly greater VI than café cats (*F* (1,28) = 6.334, *p* = 0.017). A one-sample t-test revealed that house cats’ VI was greater than 0 (*t*(13) = 2.522, *p* = 0.025) whereas that of café cats was not (*t*(15) = 1.309, *p* = 0.210).

These results indicate that only household cats anticipated a specific cat face upon hearing the cat’s name, suggesting that they matched the stimulus cat’s name and the specific individual. Cats probably learn such name-face relationships by observing third-party interactions; a role for direct receipt of rewards or punishments seems highly unlikely. The ability to learn others’ names would involve a form of social learning. New behaviors or other knowledge can also be acquired by observing other cats^31^. Recent study has reported that cats learn new behaviors from humans^32^. However, we could not identify the mechanism of learning. It is still an open question how cats learn the other cats’ names and faces.

Environmental differences between house cats and café cats include how often they observe other cats being called and reacting to calls. Contrary to human infants who are able to disambiguate the referent of a new word among many potential ones^33^, cats might not do that at least in this study. Saito et al. showed that café cats did not distinguish their own name from the name of cohabiting cats whereas household cats did so, in a habituation–dishabituation procedure^25^. We extend this finding by showing that café cats also do not appear to learn the association between another cat’s name and its face.

We also asked whether the ability to recall another cat’s face upon hearing its name was limited to conspecifics. How about human family members? In Exp.2 we used household cats and re-ran the same experiment using a family member’s name and face.

A limitation of Exp.1 was that we could not analyze the effect of the duration of cohabiting with the model cat because this differed across cats, and in some cases the information was lacking (i.e., it was hard to track the exact length of time subject and model cats lived together, as the owner quarantined cats that didn't get along with others.). We predicted that the longer the cat and human had lived together, the stronger the association between name and face would be, due to more opportunities to learn it.

## Experiment 2

The procedure in Exp.2 was almost the same as in Exp.1, but we used human instead of cat stimuli. In view of likely differential exposure to name–face relationships depending on the number of people living together (for example, someone living with a single other person calls that person’s names less often than someone living with multiple others), we took this factor, along with length of time living together, into account in the analysis.

### Materials and methods

#### Subjects

We tested 26 household cats (15 males and 11 females, mean age 5.2 years, *SD* 3.27 years) living in houses with more than two people. Thirteen cats lived with two-person families, seven with three-person families, four with four-person families, and two with five-person families. Durations of living together ranged between 6 and 180 months (*mean* 49.79 months, *SD* 41.50). We did not ask the owner to change water or feeding schedules.

#### Stimuli

The experimental stimuli were the same as Exp.1 except that we used human names and faces instead of cat names and faces, and unfamiliar voices instead of owners’ voices (mean duration 0.80 s, *SD* 0.30) (i.e., the person calling the name was never the person whose face was presented). As in Exp. 1, we used habitually used names instead of real names to ensure that the cats had the opportunity to learn on a daily basis (e.g., “mother”). All sound files were adjusted to the same volume with Audacity(R). One experimenter took the photos, face-forward and smiling, with a plain background (resolution range x = 304 to 4608, y = 340 to 3512) which were adjusted to the monitor size. If the model could not be present on the day of the experiment the owner sent a family photo by e-mail in advance. In households of more than two people, the models were decided randomly.

#### Procedure

The procedure was the same as in Exp.1.

#### Analysis

We conducted almost the same statistical analysis as in Exp. 1. One cat was tested only on the first trial because she escaped from the room. In total, 32 congruent and 27 incongruent trials were analyzed, after excluding 42 “no attention” trials. We measured duration of attending to the monitor as in Exp.1 and analyzed the data by a linear mixed model (LMM) using a lmer function in a lme4 package version 1.1.10^28^. We log-transformed the attention data to better approximate a normal distribution. We log-transformed the duration of living together to reduce variance. Congruency (congruent/incongruent), number of family members (2–5), duration of living together and interactions were entered as fixed factors, with subject identity as random factor. To clarify the effect of duration of living together, we assigned cats to two groups: those living with their humans for above-median durations were the “Long” group, and those with below-median durations were the “Short” group.

In addition to attention, we analyzed VI. Because we used data from subjects with at least one congruent—incongruent pair, this concerned 16 subjects. We ran a linear model (LM) using a lmer function in a lme4 package version 1.1.10^28^ with the number of family members (2–5) and duration of living together entered as fixed factors.

### Results and discussion

**Figure 4:**
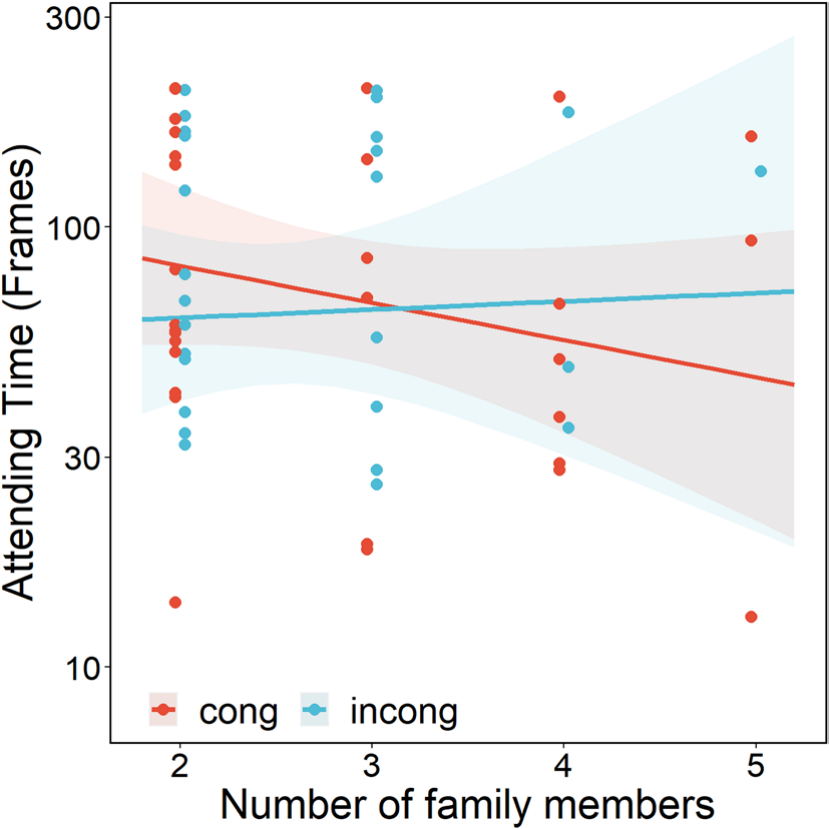
Time attending to the monitor during the face phase in Exp.2. Red points represent congruent condition; blue points represents incongruent condition. Each line represents a regression line predicted by the LMM. Each ribbon represents the 95% confidence interval. The y-axis is log-transformed.

**Figure 5:**
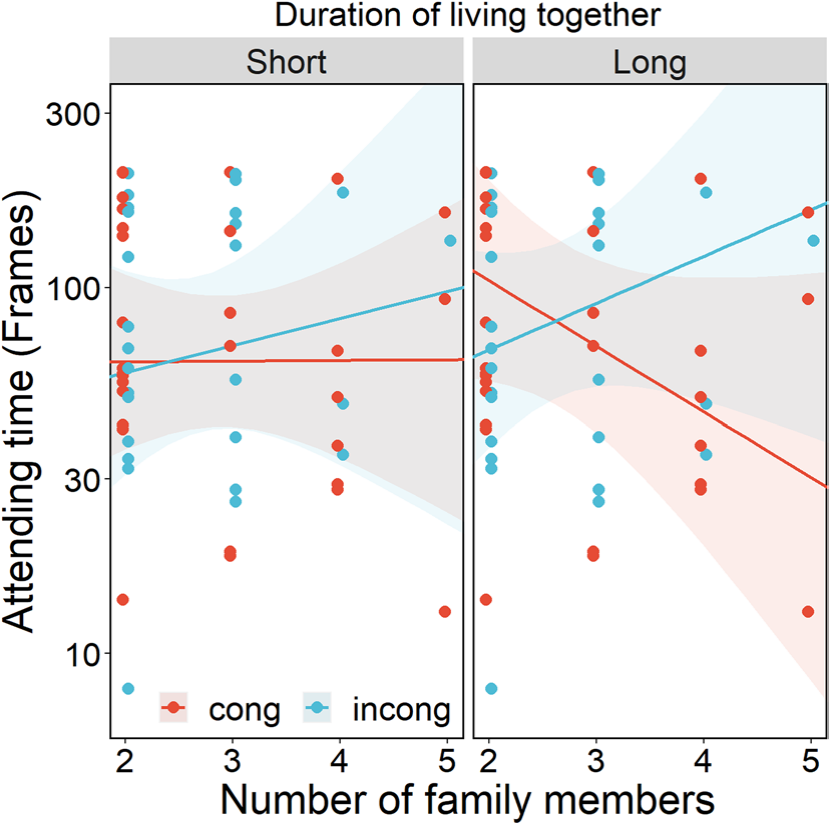
Time attending to the monitor during the face phase grouped by time living together in Exp.2. We separated time living together into short and long groups by median for convenience because we found a significant interaction of time together, number of family members and congruency. Left panel represents short group; right panel represents long group. Red points represent congruent condition; blue points represent incongruent condition. Each line represents a regression line predicted by the LMM. Each ribbon represents the 95% confidence interval. The y-axis is log-transformed.

Figure 4 shows time spent attending to the monitor according to the number of family members. The more the number of family members increased, the longer cats attended to the monitor in the incongruent compared to the congruent condition. LMM revealed significant interactions of congruency × number of family members (${\rm X}^{2}$(1) = 3.885, *p* = 0.048) and congruency × number of family member × duration of living together (${\rm X}^{2}$ (1) = 3.920, *p* = 0.047). There was no significant main effect of congruency (${\rm X}^{2}$(1) = 0.066, *p* = 0.797). Figure 5 shows attention to the monitor for each group divided by length of time living together, to illustrate the 3-way interaction. The Long group strengthened the tendency (the more family members, the greater attention in the incongruent condition), whereas the short group weakened the tendency, with fewer differences between congruent and incongruent conditions.

**Figure 6:**
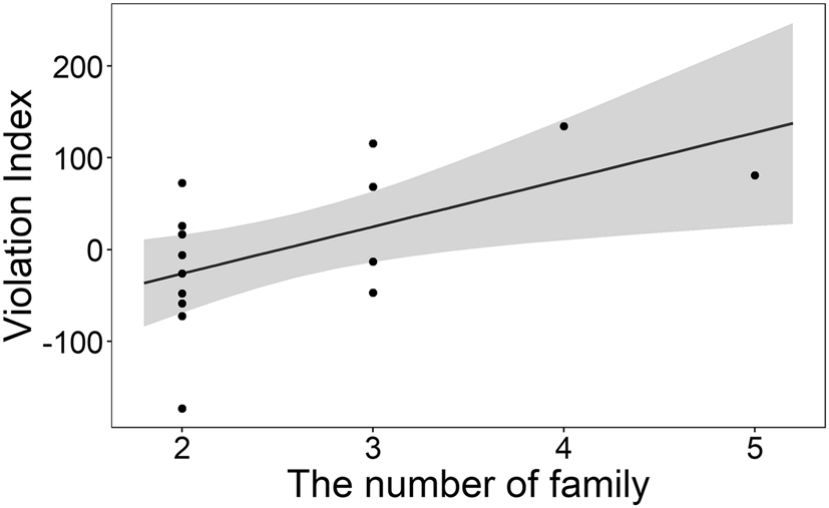
The relationship between Violation Index and number of family members. Grey area indicates the 95% confidence interval predicted by the LM.

Figure 6 shows the relation between VI and the number of family members. With increasing family size, the VI scores were higher. LM revealed a significant main effect of number of family members (*F* (1,12) = 6.522, *p* = 0.025). However, there was no significant interaction between number of family members and duration of living together.

These results suggested that not all cats predict a familiar human’s face upon hearing that name; we found no main effect of congruency. However, the interaction among congruency, number of family members and time living together indicated that attending to the monitor was affected by time spent together and family environment: the bigger the family, the more cats attended in the incongruent condition and the less they attended in the congruent condition; this was especially so for the cats that had lived longest with their human family. Our interpretation is that cats living with more people have more opportunities to hear names being used than cats living with fewer people, and that living with a family for a longer time increases this experience. In other words, the frequency and number of exposure to the stimuli may make the name–face association more likely.

### Ethical statement

This study adhered to the ethical guidelines of Kyoto University and Azabu University, and was approved by the Animal Experiments Committee of the Graduate School of Letters, Kyoto University and the Ethics Committee of Azabu University, which follows “Guidelines for Proper Conduct of Animal Experiments” by the Science Council of Japan (2006). Informed consent was obtained from all owners.

## General discussion

This study examined whether domestic cats learn that human utterances indicate specific objects in their daily life. In Exp.1, cats were presented with the face of another cat from the same household after hearing playback of either a matching or a mismatching name. Results revealed that house cats, but not café cats, attended to the monitor for longer in a name-face incongruent condition than the congruent condition. Upon hearing a cats’ name, the subjects expected the corresponding face. In Exp.2, we used human stimuli to examine whether cats also expect the face of human family members upon hearing their names. Results showed that, although not all cats attended for longer duration in the incongruent condition, the number of household members affected their responses: with more family members, cats attended for longer to the monitor in the incongruent condition. Furthermore, cats that had lived with their family for longer showed the longest durations of attention when the name–face relationship was incongruent. These results might suggest that cats might learn names from observing interactions between humans: a third-party perspective. However, it was not procedurally possible to identify the mechanism of learning in this study. Further study should clarify how cats learned the association. In summary, house cats matched at least their companion cats’ names and faces, and possibly their human family members’ names. This is the first evidence that domestic cats link human utterances and their social referents through every day experiences.

Acquisition of new signal-meaning pairs requires a high level of social cognition, such as knowing to whom others are talking and paying attention^34^. Many recent reports on social cognition in cats (see review^35^), have shown their high sensitivity to human attentional states^20–22^. These results of the present study suggest that cats might understand who is talking to whom in everyday situations, which is consistent with those studies. However, it is still unclear how cats learned the name-face association. Further study should address this point.

In Exp.1, we found a difference between household cats and café cats. Previous studies have reported several behavioral differences between these two groups^24,25,36^. In Saito et al. house cats but not café cats were shown to recognize their own name; café cats did not discriminate their own name from names of other cats living in the same environment. Whereas house cats probably learn by observing the reaction of the specific cat whose name was called, café cats are more likely to hear different names called by different guests, making such learning more difficult. Additionally, the number of cats living together might have an influence, as more cats probably means fewer opportunities to learn specific cat name-identity relationships. In our experiment, 75% of café cats tested lived in cafés holding over 30 cats (Supplementary Table S1). In fact, recent research has shown that people with larger social networks show poorer voice recognition^37^. To untangle any effects of number of cats living together and fewer opportunities to observe interactions of each cat, cats from cafés of different sizes could be tested.

In this study we did not take into account the nature of the social relationships between cats. Model cats were randomly chosen among subjects’ cohabitants, without regard to the quality or their relationship with the subject. It could be useful for further studies to examine this factor as well as the influences of experience, environment, and familiarity of model cats on cats’ learning of human utterances.

We used familiar voices as auditory stimuli in Exp.1 and unfamiliar voices in Exp.2. Cats responded more in incongruent condition in Exp.1 but less clearly so in Exp.2. Perhaps cats will generally show clearer expectancy violation effects when names are called by familiar voices. Some previous studies have shown cat-human communication effects specific to the owner, with little generalization of social cognitive abilities to a stranger^18,38^. Galvan and Vonk reported that cats differentiate between happy and aggressive expressions of their owner but not a stranger^18^. Although Saito et al. reported that cats recognized their own name even when called by a stranger^25^, this was not the case for a family member’s name, possibly due to weaker association in the latter situation. To more closely examine whether cats understand common phonetic characteristics in human utterances beyond the owner’s voice, future studies should use strangers’ voices as stimuli.

We found that cats recognize at least one companion cat’s name and possibly a human family member’s name. It might be asked what motive cats have for remembering names. One possible explanation has to do with competition. For example, a cat might receive food when the owner calls her name but not when she calls another cat’s name. The fact that humans are probably not in competition with cats might explain the weaker association between human names and faces.

In Experiment 2 we found that cats' looking behavior changed with the length of time living with a human family. However, this length was highly correlated with cat age (Pearson's r = 0.89). Because cognitive abilities develop with age, the relationship we observed may reflect an age effect. We were unable to isolate these factors in this study; this should be done in future research.

Previous studies of companion animals’ understanding of human speech have focused on “exceptional” subjects with intensive training and subsequent excellent performance in remembering names of many objects (in dogs^7,8^). By contrast, recent work revealed that “normal” dogs did not perform as impressively as “exceptional” dogs^39^. However, those studies did not clarify whether subjects had a visual image of the referent after hearing a name. Our study demonstrated that cats expect a specific face upon hearing the specific name of a companion. We conducted no training, but exploited cats’ spontaneous learning of relationships between names and faces in their everyday experiences, similar to what human children do. Further study should test whether cats are sensitive to the symbolic nature of some human utterances.

We did not control or measure affective aspects of hearing the other cat's or human’s name. Further studies along these lines should consider using emotionally neutral items and their names, removing possible affect-related influences, to shed further light on cats’ ability to link names and objects of more neutral valence.

In conclusion, house cats linked at least two conspecific housemates’ human-given “names”. They might have a cross-modally integrated concept of another cat’s name and face, similar to humans. This study differs from well-known field studies^2^ in that the stimulus utterance was not related to any urgent, potential life or death situation. A remaining question is *how* cats learn names. Language learning is known to be affected by prosodic aspects. Infant-directed speech characterized by prosodic exaggeration and lexical and syntactic simplification facilitates word learning in infants^40^. An fMRI study revealed that dog brains dissociated lexical and emotional prosodic information in human spoken words, similar to humans^9^, which might facilitate language learning. Prosodic factors might affect cats in the same way, which would be interesting for how they learn about the referential nature of human utterances. Another question concerns the evolution of this ability. Some researchers have proposed that (self-) domestication was important in human language evolution^34,41^. Future research could address this issue by working with African wildcats, or other domesticated animals such as dogs and horses.

## Supplementary Information

Supplementary Information 1.

Supplementary Information 2.

Supplementary Tables.

## Data Availability

We attached data on e-letter which contains all data we used this study.
