# Author Correction: Cats learn the names of their friend cats in their daily lives

**DOI:** 10.1038/s41598-023-40125-5

**Published:** 2023-08-10

**Authors:** Saho Takagi, Atsuko Saito, Minori Arahori, Hitomi Chijiiwa, Hikari Koyasu, Miho Nagasawa, Takefumi Kikusui, Kazuo Fujita, Hika Kuroshima

**Affiliations:** 1 Department of Psychology, Graduate School of Letters, Kyoto University, Yoshida-honmachi, Sakyo, Kyoto, 606-8501 Japan; 2 Department of Animal Science and Biotechnology, Azabu University, 1-17-71, Fuchinobe, Chuo-ku, Sagamihara, Kanagawa 252-5201 Japan; 3 Japan Society for the Promotion of Science, 5-3-1, Chiyoda-ku, Tokyo, 102-0083 Japan; 4 Department of Psychology, Faculty of Human Sciences, Sophia University, 7-1, Kioicho, Chiyoda-ku, Tokyo, 102-8554 Japan; 5 Research and Development Section, Anicom Speciality Medical Institute Inc., 2-6-3 Chojamachi 5F, Yokohamashi-Nakaku, Kanagawaken, 231-0033 Japan; 6 Wildlife Research Center, Kyoto University, 2-24 Tanaka-Sekiden-cho, Sakyo, Kyoto, 606-8203 Japan

Correction to: *Scientific Reports* https://doi.org/10.1038/s41598-022-10261-5, published online 13 April 2022

The original version of this Article contained an error in Figure 1, where an arrow for the congruent condition was missing. The original Figure 1 and accompanying legend appear below.

**Figure 1:**
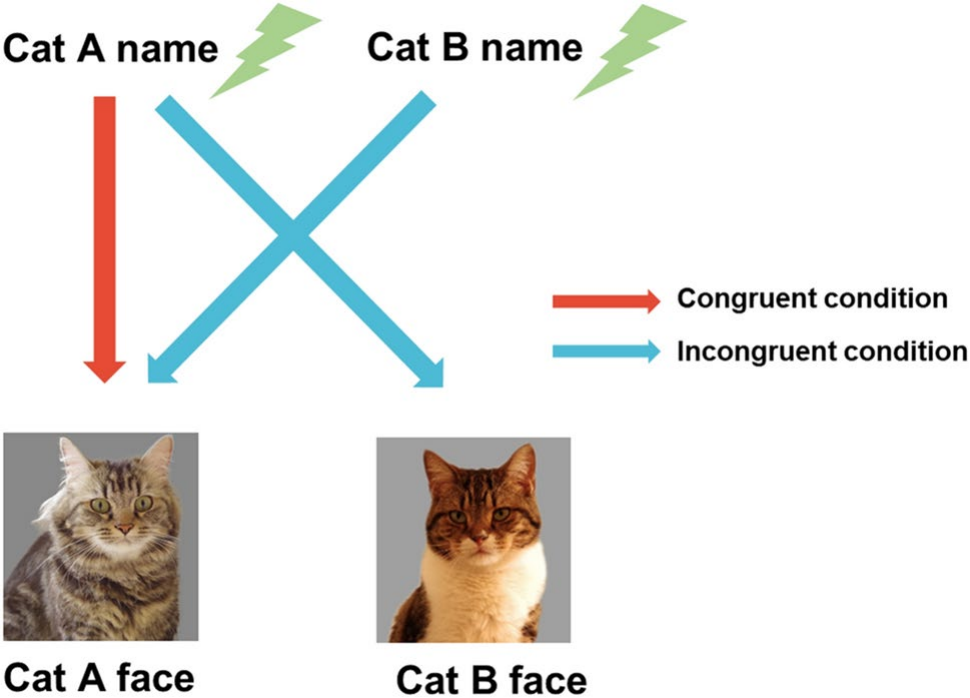
Diagram illustrating each condition in Exp.1. Two model cats were chosen from cats living with subject. The model cat’s name called by owner was played through the speaker built into the laptop computer (Name phase). Immediately after playback, a cat’s face appeared on the monitor (Face phase). On half of the trials the name and face matched (congruent condition), on the other half they mismatched (incongruent condition).

The original Article has been corrected.

