# Supplementary material for: Cats learn the names of their friend cats in their daily lives
Source: Sci Rep. 2022 Apr 13;12:6155. doi: 10.1038/s41598-022-10261-5 (PMC9007945; doi:10.1038/s41598-022-10261-5)
Supplement: Supplementary file 3 — Supplementary Tables. [file 41598_2022_10261_MOESM3_ESM.docx]

Supplement

Table.S1

Subject information in Exp.1

| S | Place | Living | Number of cats living together |
| --- | --- | --- | --- |
| S1 | time | cafe | 10 |
| S10 | pasya | cafe | 6 |
| S11 | pasya | cafe | 6 |
| S12 | nyan | cafe | 7 |
| S13 | nyan | cafe | 7 |
| S14 | time | cafe | 10 |
| S15 | time | cafe | 10 |
| S16 | koshien | cafe | 30 |
| S17 | koshien | cafe | 30 |
| S18 | time | cafe | 10 |
| S19 | koshien | cafe | 30 |
| S2 | time | cafe | 10 |
| S20 | koshien | cafe | 30 |
| S21 | koshien | cafe | 30 |
| S22 | koshien | cafe | 30 |
| S23 | koshien | cafe | 30 |
| S23 | koshien | cafe | 30 |
| S24 | koshien | cafe | 30 |
| S25 | koshien | cafe | 30 |
| S26 | koshien | cafe | 30 |
| S27 | koshien | cafe | 30 |
| S28 | koshien | cafe | 30 |
| S29 | nodai | cafe | 3 |
| S3 | time | cafe | 10 |
| S4 | wannyan | cafe | 20 |
| S5 | wannyan | cafe | 20 |
| S6 | wannyan | cafe | 20 |
| S7 | koshien | cafe | 30 |
| S8 | koshien | cafe | 30 |
| S9 | koshien | cafe | 30 |
| S30 | wakayama | house | 7 |
| S31 | wakayama | house | 7 |
| S32 | wakayama | house | 7 |
| S33 | wakayama | house | 7 |
| S34 | pure | house | 10 |
| S35 | pure | house | 10 |
| S36 | otsu | house | 3 |
| S37 | otsu | house | 3 |
| S38 | otsu | house | 3 |
| S39 | abi | house | 6 |
| S40 | abi | house | 6 |
| S41 | nagaoka | house | 3 |
| S52 | nagaoka | house | 3 |
| S54 | hikone | house | 3 |
| S55 | aichi | house | 15 |
| S56 | aichi | house | 15 |
| S57 | murata | house | 4 |
| S58 | murata | house | 4 |
| S59 | murata | house | 4 |

Table.S2

Subject information in Exp.2

| S | Breeding time(Months) | Number of family | model1 | model2 |
| --- | --- | --- | --- | --- |
| S1 | 6y3m | 3 | Daughter | Husband |
| S2 | 1y | 3 | Daughter | Husband |
| S3 | 3y3m | 2 | Wife | Husband |
| S4 | 2y5m | 2 | Wife | Husband |
| S5 | 6y6m | 3 | Daughter | Husband |
| S6 | 5y3m | 2 | Wife | Husband |
| S7 | 8y | 2 | Wife | Husband |
| S8 | 1y | 5 | Daughter | Wife |
| S9 | 5y5m | 5 | Daughter | Wife |
| S10 | 3y | 3 | Daughter | Husband |
| S11 | 5y | 2 | Wife | Husband |
| S12 | 15y | 3 | Daughter | Wife |
| S13 | 9y | 3 | Wife | Husband |
| S14 | 6m | 2 | Wife | Husband |
| S15 | 2y3m | 3 | Son | Husband |
| S16 | 6y | 2 | Daughter | Wife |
| S17 | 6m | 2 | Wife | Husband |
| S18 | 3y2m | 4 | Daughter | Wife |
| S19 | 3y2m | 4 | Daughter | Wife |
| S20 | 1y6m | 4 | Son | Wife |
| S21 | 2y4m | 4 | Daughter | Wife |
| S22 | 3y | 2 | Wife | Husband |
| S23 | 4y | 2 | Wife | Husband |
| S24 | 2y4m | 2 | Wife | Husband |
| S25 | 2y | 2 | Wife | Husband |
| S26 | 2y4m | 2 | Wife | Husband |

Legend

Exp.1_dataset: There is a dataset that we used analysis for Exp.1 .

Exp.2_dataset: There is a dataset that we used analysis for Exp.2.
